# The Bubble of Normalisation: A Qualitative Study of Carers of People With Dementia Who Do Not Seek Help for a Diagnosis

**DOI:** 10.1177/08919887211060018

**Published:** 2021-12-24

**Authors:** Michelle Parker, Sally Barlow, Juanita Hoe, Leanne M. Aitken

**Affiliations:** 1 Division of Nursing, 4895 City University of London, London, UK; 2 School of Health Sciences, 4895 City University of London, London, UK; 3 School of Nursing & Midwifery, Griffith University, Australia

**Keywords:** dementia, diagnosis, carer, stigma, normalisation, help seeking

## Abstract

**Objective:**

Improving dementia diagnosis rates are a key feature of dementia strategy and policy worldwide. This study aimed to explore the experience of carers of people diagnosed with dementia during or following a hospital admission in order to identify factors that had prevented them from seeking help beforehand. Semi-structured interviews were conducted with 12 informal carers including adults caring for a parent, a friend or a spouse diagnosed with dementia between 2010–2019, following an acute hospital admission for a physical health problem, having not sought help previously.

**Main Findings:**

Carers created a ‘bubble of normalisation’ around themselves and the person living with dementia (PLWD) to reject the label of dementia and protect the PLWD from a loss of independence, discrimination and prejudice they felt would be the result of a diagnosis. Carers struggled to talk to the PLWD about dementia reinforcing denial and stigma. Post-diagnosis carers felt unsupported and questioned the value of diagnosis.

**Principal Conclusions:**

Stigma related to images of dementia as a disease that takes away independence and identity prevented discussion about dementia between carers and the PLWD. A lack of open discussion about memory concerns between health care professionals and carers also served to delay diagnosis.

## Introduction

Worldwide, it is estimated that over 50 million people are living with dementia.^1^ Yet it is estimated that up to 50% of people in high-income countries and 95% of people in low and middle-income countries have not received a formal diagnosis.^2^ In 2017, the World Health Organisation set an ambitious target – for 50% of people estimated to have dementia to have received a formal diagnosis in a minimum of 50% of countries worldwide by 2025.^3^ Access to dementia diagnostic services and the standards of diagnostic data collected and monitored vary across the globe, resulting in difficulties in achieving and verifying diagnoses.^4^ However, where dementia diagnostic services are widely available, a deeper understanding of why people do not seek help is needed. Participants in studies exploring delays and barriers to diagnosis do eventually seek a diagnosis, and very little is known about those who remain undiagnosed.

National dementia strategies, plans and policies advocate for a timely diagnosis, one made when the person or their family ‘express concerns and have a need for advice, treatment or support’ (p5).^2^ Despite this intention, the term ‘timely diagnosis’ is often used to refer to a diagnosis made early in the disease process. In the absence of a cure or disease-modifying therapy, the potential benefits to people with dementia and their carers of a diagnosis early in the disease process include reduced anxiety and uncertainty about symptoms, ability to plan for future care and support and anticipate future problems.^5^ There is evidence to suggest non-pharmacological interventions such as cognitive stimulation can produce short term improvements in cognitive function and that support interventions for persons living with dementia (PLWD) and carers can improve quality of life and delay institutionalisation.^2^ However, such interventions are often only accessible following a formal diagnosis. The pathway to a dementia diagnosis is recognised as being complex and involving an often cyclical process of noticing, appraising and normalising symptoms until a point is reached at which this can no longer be maintained and help seeking for a diagnosis begins.^6-8^ For many people this can be a lengthy process, with studies suggesting an average of between 4 months and over 3 years between first noticing a problem and seeking help.^9-11^ A recent review of barriers and facilitators to help seeking for a dementia diagnosis from the perspective of carers and PLWD suggests multiple barriers often exist for PLWD and carers, with more than one facilitator required to overcome these.^12^ Potential barriers include denial, stigma and fear, a lack of knowledge about dementia, normalisation of symptoms, lack of informal network support, a desire to preserve autonomy of PLWD, a lack of perceived need for a diagnosis and the impact on carers.^12^ Practical barriers including knowing where and how to seek help, costs related to assessment and language barriers were also noted in some countries.^12^ Physician and health system related barriers to diagnosis are also frequently reported, including a lack of confidence and ability in recognising and diagnosing dementia in primary care, a lack of time and tools to assess people in primary care and the availability of specialist diagnostic and post-diagnostic services.^13-16^ Physician’s concerns about the effect of stigma on PLWD following diagnosis and their own stigma related to dementia is also recognised as a barrier to diagnosis.^13-16^

Although stigma related to dementia is less well researched than stigma related to mental illness, for example, the association between stigma and dementia is prevalent worldwide.^1^ More negative emotions than positive or neutral emotions towards dementia are reported in studies involving lay public samples, although when compared to other illnesses, levels of stigma related to dementia are often lower.^17,18^ In other studies, PLWD and carers described perceived public stigma from seeing negative images in the media resulting in feelings of low self-esteem, shame, anxiety, fear, frustration and loss of confidence.^17,18^ The consequences of this were denying or concealing their diagnosis, social withdrawal and reduced help seeking.^17,18^ It is also possible that stigma plays a more significant role in the prevention of help seeking for dementia diagnosis than is currently supported with evidence. However, accessing a group of people in the community who are not seeking help, to understand their experiences, diagnosis wishes and support needs, is challenging.

In the UK, around two thirds of people living with dementia have a formal diagnosis and initiatives in primary and secondary health care settings have been aimed at increasing formal diagnosis rates.^19–21^ These included a case finding scheme introduced in England in 2012 to identify people with suspected dementia following emergency admission to an acute hospital setting with a physical health problem.^21^ The scheme provided an opportunity to identify undiagnosed dementia in people who had not sought help from a primary care physician.

Help seeking for a dementia diagnosis is most often initiated by a carer or close family member of the person with symptoms.^7,8^ Carers play a key role in helping the person diagnosed to retain or create a new identity post-diagnosis as well as maintain independence, whilst also adjusting to their own feelings of loss and increased responsibility.^22^ As such, exploring the experiences of carers in the pre-diagnosis period can help develop a better understanding of why some people may delay or not seek help for a diagnosis. Carers or close family members of people identified as having undiagnosed dementia on admission to hospital present an opportunity to discuss the experiences and diagnosis wishes of a group of people who are not actively seeking help for a diagnosis. As part of a larger mixed methods exploration of what prevents help seeking for a dementia diagnosis, this qualitative study aimed to explore the experience of carers of people diagnosed with dementia during or following a hospital admission in order to identify factors that had prevented them from seeking help beforehand.

## Methods

Methods are reported according to the Consolidated Criteria for Reporting Qualitative Research guidelines.^23^ Ethical approval for the study was granted by the NHS Research Ethics committee and Health Research Authority in England (IRAS 208826).

### Study Design

This qualitative study originally aimed to interview both carers and the person living with dementia, and ethical approval was gained to include people living with dementia who lacked capacity to consent to participate in an interview. To be eligible for inclusion, participants needed to have had a diagnosis of dementia initiated during or following an acute hospital admission for a physical health problem or be the carer of the person diagnosed. The diagnosis included any sub-type of dementia and any level of severity. Participants needed to have been informed of the diagnosis and speak English sufficiently to be able to participate in an interview. If the person or carer had sought help for their memory problems in primary care and this had resulted in a diagnosis, they were not eligible for inclusion. Unfortunately, no people living with dementia were recruited for the study. There are recognised challenges to recruiting people living with dementia to research studies, particularly when relying on gatekeepers in clinical practice to identify participants.^24,25^

### Procedures

**Figure 1.**
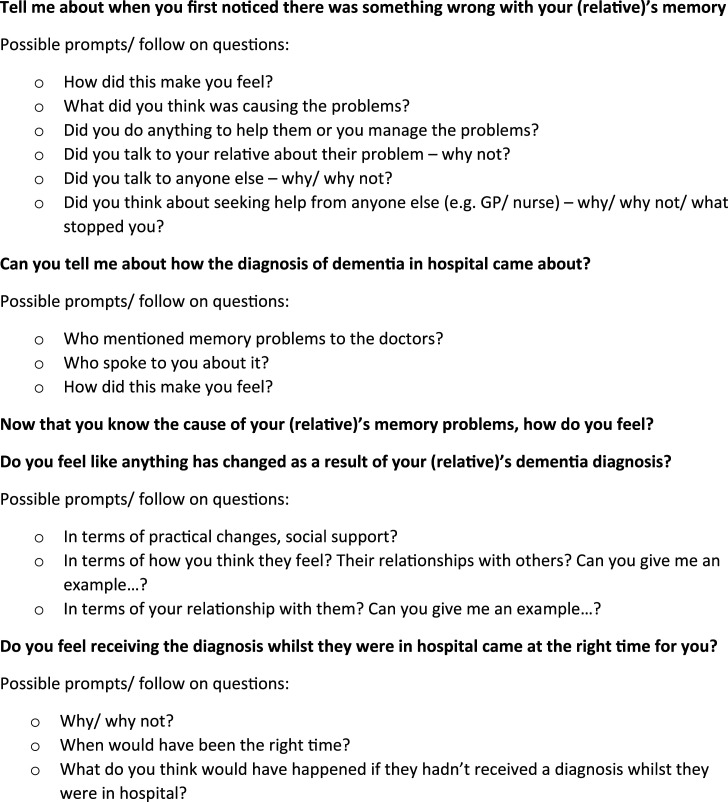
Topic guide for interviews.

Purposive sampling was used and recruitment to the study was challenging. Participants were initially recruited through the dementia teams of a large urban acute hospital in inner London following the diagnosis of a person with dementia. Only a small number of carers (n = 4) were recruited in the first 12 months. In an effort to increase recruitment, a second acute hospital trust was accessed, and the study was registered on Join Dementia Research (JDR). Join Dementia Research is a National Institute for Health Research (UK) service that allows people to register their interest in participating in dementia research and be matched to suitable studies. Subsequently a further 8 carers were recruited, all through JDR, resulting in a final sample of 12 carers. Participants were given information about the study either by nursing staff in the hospital dementia teams or were emailed information about the study by the researcher (MP) if identified as a carer for a PLWD on the JDR website. Interested participants were contacted by the researcher (MP) by telephone and email to discuss the study and arrange a face to face interview. The interviewer (MP) had not met any of the participants prior to interview. It is not known how many carers were given information by the hospital dementia teams, but 58 carers expressed an interest in the study from the JDR website. Of these only eight carers were eligible to participate, as the diagnosis of dementia had been initiated in primary care for the other 50 carers. Sampling continued until data saturation was reached, that is, when no new information was being heard.^26^ Semi-structured interviews were used as the most appropriate method to gain an in-depth understanding of carers’ experiences. A topic guide for the interview (Figure 1) was developed, discussed and reviewed with carers at a local Alzheimer’s society memory café prior to ethical approval. Interviews took place between April 2017 and November 2019. Carers recruited into the study were interviewed once in a setting of their choice, with most interviews taking place in carers’ homes. One carer was interviewed at the hospital, two at their workplace and two in public places. One carer was accompanied by another family member who did not participate in the interview. An explanation of the study and the opportunity to ask questions preceded the carer giving written informed consent to participate. Interviews were audio-recorded and carers were offered the opportunity to receive a copy of the interview transcript. Interviews ranged from 24–73 mins in length.

### Data Analysis

Interview transcripts were analysed using an inductive, latent thematic analysis approach.^27^ This approach is considered appropriate as a method which can be used to both ‘reflect reality and to unpick or unravel the surface of reality’ (p82).^27^ One author (MP) read and re-read the transcripts while listening to the audio recordings to familiarise herself with the data and then began coding transcripts. NVivo 12 Pro software^28^ was used to facilitate coding. Codes were reviewed and collated into potential themes within 3 organisational categories – pre-diagnosis, diagnosis and post-diagnosis to reflect the contextual journey of the carers. Although an inductive approach to analysis was used, the author (MP) acknowledges her prior experience of working with carers and PLWD diagnosed during an acute hospital admission and how this may have influenced the coding and analysis process. A second author (SB) with a psychology background reviewed a quarter of the transcripts and coding, and all themes were discussed and debated between the authors and reviewed and refined as necessary.

## Findings

### Participant Characteristics

The sample of 12 carers consisted of 10 adults caring for a parent (9 daughters and 1 son), one female friend carer and one female spouse carer. All carers lived in the greater London area. The people with dementia they cared for were diagnosed between 2010–2019 and were all aged in their 70s to their 90s at the time of diagnosis. Four people with dementia had died at the time of the interview. In the pre-diagnosis period, seven of the PLWD were living alone, 3 lived with the carer who was interviewed and two lived with a spouse who was not the main carer interviewed.

### Overview of Themes

**Figure 2.**
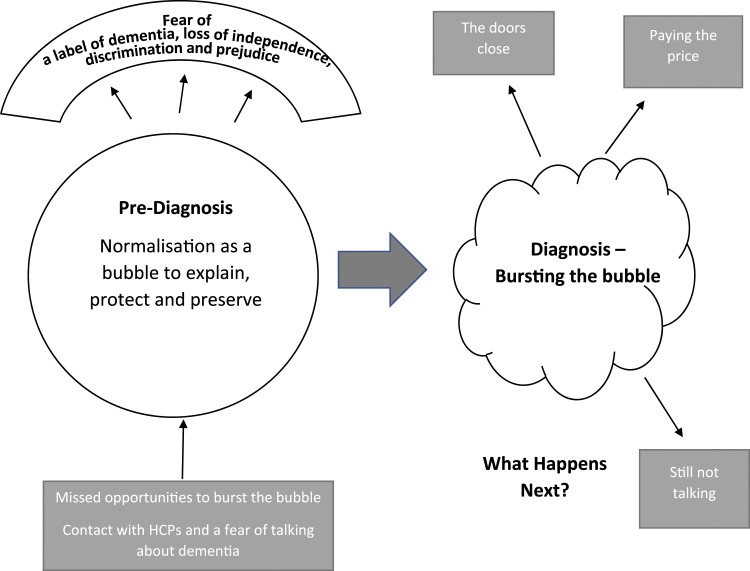
Visual representation of themes from pre-diagnosis to post-diagnosis.

Four themes represent the experience of carers from first awareness that something was wrong to post-diagnosis (Figure 2). Two themes represent the pre-diagnosis period, each with two sub-themes. The first theme *Normalisation as a bubble* describes a ‘semi-transparent barrier’ that carers construct around themselves and the PLWD rather than seek help. Within the bubble, the sub-themes *Explaining to normalise* and *To protect and preserve* describe the experience of carers as they navigate the increasing difficulties of the PLWD. The second theme *Missed Opportunities* with the sub-themes *Fear of talking about dementia* and C*ontact with health professionals* describe the missed opportunities there were to seek help in this period. The diagnosis experience for the carer is represented by the theme *Diagnosis - bursting the bubble*. The final theme of *What happens next*, with 3 sub-themes – *The doors close*, *Paying the price* and *Still not talking*, describe experiences of carers as they re-negotiate their lives after the diagnosis. Additional illustrative quotes for all themes can be found in the supplementary material.

### Theme 1: Normalisation as a Bubble

The first theme in the pre-diagnosis period describes how through a process of normalisation carers created a bubble around the PLWD, themselves and their lives. Within this bubble, the sub-theme of *‘Explaining to normalise’* describes how carers gradually realised that the changes they were witnessing in the PLWD were more than could be attributed to ageing or physical and mental health problems. They sought alternative explanations, which might include dementia, but could reject this as a possibility. The second sub-theme – *‘To protect and preserve’* describes how carers used the bubble of normalisation to protect themselves and the PLWD from a possible dementia diagnosis and to preserve the PLWD’s independence.

### Explaining to Normalise

Carers sought to explain the changes they were seeing in the PLWD in order to normalise what was happening. Most carers could recognise, with hindsight, when the person they cared for started to have difficulty and reported memory problems as first symptoms, but also behavioural and personality changes or problems with self-care.

Many carers expressed an expectation that forgetfulness came with old age and that some cognitive decline would accompany increasing physical frailty. Most of the PLWD had additional physical or mental health problems, and while some carers attributed the changes they saw directly to these conditions, others felt that these masked cognitive changes:

“She’s always had mental issues. And it took a long time I think for me to distinguish between those and this new thing.” (Carer 5)

However, for another carer attributing these early changes to ageing and the physical problems of the PLWD was easier to do, than accept that the changes might be dementia,

“I thought it’s a bit more, old age, she’s forgetful, her diabetes. I never really, the dementia was on the edge, but I never really went there too much.” (Carer 10)

At this point the carer feels there are legitimate alternatives they could attribute the changes to, so can resist the possibility of dementia. But as time went on this became more difficult for many carers. Although changes in personality could be attributed to ageing, for many there was a point of recognition that something was not right and carers began looking for other explanations.

Although the majority of carers were not living with the PLWD in the pre-diagnosis period, only one carer was unaware that the PLWD was having difficulties. But some carers struggled to explain the behavioural symptoms they saw from within their existing frame of knowledge, so constructed their own explanations as a way of normalising the behaviour, for example:

“the names that she used to call me was like one of her sisters, and then again I thought maybe overnight you know when you’re sleeping and thinking about things, maybe her sisters came into her mind.” (Carer 6)

Over time, more than half of the carers considered that the symptoms they were witnessing might be dementia. A few carers had direct previous experience of dementia, either through work or members of their family. Others had enough awareness of dementia to consider it a possibility for the person they cared for and although some recognised it was a problem that required help, this was not enough to initiate the help seeking process. Others compared what they knew about dementia, to the changes they were witnessing, sometimes making downward social comparisons (comparisons to others considered to be less fortunate than oneself) to the relatives of friends who had dementia or images of dementia they had. Although these comparisons may have been misplaced, they helped some to conclude that dementia was not the problem.

“a friend of mine, their mum is three days younger than my mum, …but she’s repeats and repeats, …and it’s all the time, but mums not like that.” (Carer 3)

Such misplaced comparisons possibly resulted from a lack of knowledge, as memory problems are often the only symptoms referred to in awareness campaigns, but if the carer was looking to reject dementia as a possible explanation, such comparisons would help them to do this. Others sought advice from friends and family, which in most cases reinforced the attribution of problems to the ageing process. Although few carers spoke explicitly of denial, they relayed negative views of dementia, the progressive nature of the disease and the impact of it on the person, which influenced their desire to normalise the changes they saw rather than formally seek help.

### To Protect and Preserve

Carers used the bubble of normalisation to preserve and protect the way of life for the PLWD and themselves, but it also served to reinforce denial that dementia might be the cause of the problems.

For many carers the process of normalisation was a conscious one. They were aware that the PLWD was experiencing difficulties and used normalisation as a way of staving off fear about what the future might hold:

“my head would think, oh my God, if it is dementia… she’d always say,…don’t ever put me in a care home,…So in my head I knew that eventually if it came to that, that’s where we would be. So… my own mind was like, just let’s just keep it in the day, she’s all right for today, we’ll deal with it as it gets, whatever happens we’ll deal with it.” (Carer 10)

Despite awareness that dementia may be the cause of her mother’s problems, the carer tried to balance her mother’s wishes for independence, with her own belief that having dementia inevitably meant institutionalisation. The fear of making such life changing decisions on behalf of her mother led her to deal with the problems day by day, to protect her mother from her own fear (‘don’t ever put me in a care home’) and keep life as it is, for as long as she can. This was a conflict for many carers as they sought to balance the independence of the PLWD with their increasing need for support. Carers managed this conflict by providing the care themselves if they could and put in place practical measures to support the person and mitigate safety concerns, for example, moving the bed downstairs, moving to sheltered accommodation or moving to be closer to family members. Carers expressed a sense of duty to protect the person, supporting them practically to take ‘the fear out of it’.

Another carer described constructing, ‘a semi-transparent barrier, you know one of the ones where you think if you just live day by day things will resolve, resolve themselves right’. This creates a vision of normalising as a ‘bubble’, in which the carer, the PLWD and their way of life are protected and preserved and one that only the diagnosis of dementia could burst.

For some carers it appeared that a formal diagnosis or labelling of the problems as ‘dementia’, somehow made the problems more real. Although many carers appeared aware of the progressive nature of the disease, by not seeking a diagnosis, they were delaying a process that would not begin until a diagnosis was made. This was particularly stark in one carer’s experience, when as his mother received a diagnosis towards the end of her life, he expressed a sense of relief that she was ‘not going to live through dementia’, despite having had quite severe behavioural and psychological symptoms of dementia for a long time.

For another carer there was a fear that a label of dementia would mean her mother could no longer stay in the supported accommodation she lived in, and this carer actively resisted requests from staff at the accommodation to seek help for a dementia assessment. In discussing this, the carer describes how

“you’re fighting a lot of negative stereotypes about old people…and if you add the label of Alzheimer’s on top of that… you really are up against an awful lot of prejudices” (Carer 5).

A diagnosis of dementia had the ability to add to the prejudices and discrimination that older people in society already faced. Suggesting that the process of normalising may be used not only to protect the person from a diagnosis which will change their life but also to protect them from the reactions of others and societal prejudices towards people with dementia.

The fear and reactions of others towards PLWD could make maintaining normalisations and protecting the person more difficult for the carer. One PLWD in sheltered accommodation encountered discrimination from other residents, as seen in the extract below,

“people that had been her friends, they turned on her because they found it disturbing… they would get upset by it and they were going we can’t cope because I’m old, I don’t want this lady giving me her problems and it was awful because in a way she got bullied… it was also isolating her more.” (Carer 2)

As other residents were urging the carer to seek help, the carer was torn between respecting the autonomy of the person, who had expressed she did not want any help from social services and protecting them from the discrimination of others.

Maintaining normalisations became increasingly difficult for carers over time, as PLWD needed increasing support with activities of daily living, had instances of getting lost, forgetting to take medication and burning saucepans. Despite the increasing burden, help seeking for a diagnosis was not an option carers considered, instead this was outweighed by their desire to protect the person and maintain the bubble of normalisation.

### Theme 2: Missed Opportunities

The second theme in the pre-diagnosis period is ‘Missed Opportunities’. The two sub-themes – *‘Fear of talking about dementia’* and *‘Contact with health professionals’* represent missed opportunities to seek help for a diagnosis.

### Fear of Talking About Dementia

Fear of talking about dementia prevented carers and people with dementia from discussing seeking help, thereby maintaining the bubble of normalisation. Only a small number of carers spoke to the PLWD about their concerns and in all cases their concerns were dismissed by the PLWD. Carers attributed this to the pride and independence of the person and to their personality:

“[He] would never discuss whether he would be cremated or buried, for example. He just wouldn’t talk about that sort of thing…So…it wasn’t surprising that he didn’t want to have a test because he just, he didn’t like talking about things like that.” (Carer 7)

Likening the discussion of dementia to the discussion of death, suggests fear. There was a belief that even having a test for dementia equated to talking about death, and the carer felt this fear was understandable, so the difficulties were not spoken of again and the bubble of normalisation was maintained.

Most carers did not discuss their concerns with the PLWD. In defending their decisions not to talk, carers again spoke of the independence and personality of the PLWD:

“she’s always been so fiercely independent…I don’t think she’d have entertained it at all. Not at all. And she’d have probably been really, really angry with me for bringing it up.” (Carer 11).

“she’s very protective of herself and very ‘nobody knows me better than myself thank you very much’. And we could never have those discussions.” (Carer 5)

But they also evoked fear of not wanting to damage the relationship they had with the PLWD, by making them angry or changing the relationship they always had. Such relationships, based on autonomy and respect, may have been valued by the carer over any benefit they perceived from suggesting seeking help.

By respecting what they thought the PLWD would want, the carer’s own desire to maintain the status quo could be enabled. Very few PLWD expressed their concerns to carers in this study. But when they did, carers were dismissive and sought to reassure them that nothing was wrong, despite most having concerns that the person might have dementia.

“I’d go, mum, it’s all right, it happens to all of us, perhaps you’re a bit tired or, you’ve got a lot on your mind and just try and put her mind at rest.” (Carer 11)

Two carers described how the PLWD had recorded their concerns in a diary, which the carer was not aware of until after the death or diagnosis of the person. On reflection, one carer wished she had possessed ‘the tools to talk to both of my parents and really get them to open up about the severity of their symptoms…I think it would have pushed me to do more of that diagnosis’. (Carer 9).

The opportunities to have a conversation that might lead to seeking help were missed, and for most carers, the feelings and wishes of the PLWD were unknown. This unspoken truth served to reinforce a denial of the possibility of dementia and the bubble of normalisation.

### Contact With Health Professionals

There were missed opportunities either for the carer to seek help when they came into contact with health care professionals (HCPs) or for an assessment and diagnosis to be initiated by HCPs. Many of the people with dementia were already seeing their General Practitioner (GP) regularly for other physical health problems, and in most cases, they were attending these appointments alone. Carers respected the person’s wish to attend alone and as such did not know whether the person had expressed concern about their problems to their GP or if their GP had expressed concern to them. Some carers felt it was likely that the person’s GP was aware of problems:

“He was being seen by the GP fairly regularly for other things, because he’d had three hips and a knee and he’d had a heart valve and so he was being seen reasonably frequently…I’m sure the GP noticed differences. So, I think it would have been that the GP could have suggested a diagnosis.” (Carer 7)

Evidently the GP was very familiar with the person, and there was an expectation from the carer that the GP should have made a diagnosis. This was a sentiment echoed by other carers and one carer felt that ‘what would really have been useful, would have been the doctor [GP] to say to me, look, your mother’s got this, and for her to say it to my mother as well’.

Both carers might have welcomed a diagnosis if the GP had initiated it at this point. In this way, the carer would have been absolved of making decisions to seek help against the wishes of the PLWD or have a difficult conversation with the person about their concerns. The GP would have assumed responsibility and taken it out of their hands.

In the same way, physical problems could overshadow cognitive problems when carers first noticed changes, physical health problems continued to dominate carer’s contact with health services. However, their contacts with primary and secondary care services were limited to managing individual problems, for example, a urinary tract infection or a fall. Despite having their own concerns, carers did not use these opportunities to seek help, even when they were aware that the person had brief memory assessments in hospital. Some may not have realised that these were appropriate times to express their concerns, but for others, they were not yet ready to take the next step and pursue a diagnosis:

“they did some sort of MRI or PET scan…they saw that there was a certain loss. But it didn’t trigger anything in us because you know we were ignorant… but somehow there’s a barrier beyond which none of us wants to go.” (Carer 8)

The carer recognised that their lack of knowledge might be the contributing factor to not asking for more information about the scan results or seeing this as an opportunity to express her concerns. They also acknowledged that maybe they did not want to know and did not want to cross ‘a barrier’ that may lead to a dementia diagnosis. Making excuses for the PLWD’s poor performance on memory tests was also easy when the PLWD’s first language was not English.

For another carer, there was again an expectation that HCP’s would initiate the diagnosis:

“they can see the signs, so why don’t the medical people tell me that they can see the signs, what are they frightened of telling you.” (Carer 3).

This carer suggested that the stigma of dementia extended to HCPs and described it as,

“it’s almost like it’s a taboo subject… do you think that people might think well you don’t wanna know or something? You don’t wanna know that your mums got dementia or you don’t wanna know that your dad’s got Alzheimer’s” (Carer3)

Stigma faced in clinical settings represented a real conflict in carers, who in their process of normalising had avoided talking about or seeking help for a diagnosis of dementia, but with hindsight wished HCPs had taken the opportunity to make a diagnosis. A few PLWD had been assessed by a memory clinic or community memory team at some point, although not diagnosed with dementia. Carers seemed ambivalent as to the outcome of these assessments and sometimes dismissive of them, ‘I just think he was looking for a bit of sympathy’. This may reflect the struggle they had in discussing the outcomes of memory assessments with the PLWD and what these might mean for the future. It may also represent the carers own fears and a wish not to confront the possibility of a future in which their loved one has dementia.

For a few carers their experience of contact with health care services was that of a “fragmented system” as the person moved between primary and secondary care services or between physical health, mental health and social services.

“They just, I think they might have assumed that she’d already been diagnosed or she was about to be diagnosed or things were in hand”. (Carer 5)

Carers recognised the missed opportunities to seek help with hindsight, but again there was an expectation that HCPs would have initiated this. When one carer attempted to seek help the system itself proved a barrier, with each service seemingly reluctant to be the one that initiated the diagnosis:

“social services push you to the mental side, because she mentally needs to be assessed…but he [mental health worker] said no social services have to deal with it first…so they just both bashed her backwards and forwards, no-one was interested really” (Carer 2).

Contact with HCPs seemed to be full of missed opportunities, not only for the carer to express their concerns but also for HCPs to acknowledge their concerns and ask the person and/or carer if they wanted an assessment for diagnosis. Carers appeared to expect HCPs to initiate the conversation and assessment, but at the same time HCPs might have expected carers or the PLWD to initiate the conversation if they had concerns or needed help.

### Theme 3: Diagnosis - Bursting the Bubble

For all carers a point came where their bubble of normalisation was burst, and a diagnosis of dementia was made. Most of the diagnoses took place in hospital with a small number happening in the community following discharge. A few carers played a role in initiating the assessment and diagnosis during or following the hospital admission. These carers used the admission as an opportunity to ask for symptoms to be investigated or knowing that investigations had taken place, asked for these to be followed up either whilst the PLWD was still in hospital or after discharge:

“I knew that they had done a scan. So, I asked if, could they look at it and tell me whether they thought that he did he have dementia.” (Carer 7)

For a carer whose father had dismissed her concerns and refused to talk about what was happening, the admission to hospital and investigations that took place gave her a legitimacy that she had not previously felt, to seek help on his behalf.

The decision to assess and diagnose the person happened without the carers involvement in some cases and sometimes without them being aware. The diagnosis was disclosed over the phone by a hospital psychiatrist or doctor from the memory clinic to some carers. For others, it was in person, but some carers appeared quite vague as to who exactly had told them, either because of the passage of time or because it came amongst many conversations they had with doctors during the admission. One carer only became aware of the diagnosis from reading the PLWD’s discharge notes.

Many carers suspected the person might have dementia but had explained and normalised what was happening over a significant period of time. However, on receiving the diagnosis they expressed a range of emotions. Some were relieved because ‘we needed to know what was the matter with her’ and because ‘I believed it anyway, but it’s always nice to have something confirmed’. For others, there was fear and disbelief:

“I was really frightened…everything they were saying I wasn’t accepting it because I wanted my mum to be the mum I knew.” (Carer 6).

For this carer, the naming of the problem, the label of dementia, had the power to change who her mother was (‘I wanted my mum to be the mum I knew’), despite her having experienced quite severe cognitive problems for a long time. In the earlier theme of *To protect and preserve,* the label had the power to precipitate the decline, at this point the label of dementia had the power to change the person in the eyes of this carer and for others was seen as a label they would want to hide or one which was only made to facilitate discharge. One carer’s reaction to the diagnosis conveys his sense of anger, and then his resignation as his attempts to protect his mother from a label of dementia are unravelled:

“I thought how dare you say…[she has dementia], I’m her son I don’t think she…[has dementia]”…“and so I had to concede, I didn’t want her to have dementia, be labelled with dementia” (Carer 1)

In addition to changing the person with dementia, one carer described how the diagnosis had the power to change her:

“you’re just an ordinary citizen before it’s happens aren’t you…I mean you just you become a different person or at least you see the world in a different way.” (Carer 8)

Carers reactions to the timing of the diagnosis were mixed. Some felt that the hospital setting was not appropriate, others wished the diagnosis had been made earlier and reflected on what they might have done differently, whilst others felt the timing was right and that knowing earlier may have been worse:

“I don’t know if it would have made any difference having that diagnosis earlier. I think it might have made it actually a little bit worse because I would have been keeping her at home. I would have tried to keep her at home as long as she could, because I think once someone goes into a care home it takes away a lot of their own individuality.” (Carer 10)

Again, the importance placed on the independence of the PLWD is reflected in the carer’s comment above, as they equate having an earlier diagnosis with an increased possibility of not being able to support the PLWD at home and earlier institutionalisation.

This realisation that the world was changing, for the person and for the carer, was also reflected in the way other carers reacted to the diagnosis, ‘I felt sad for her, because I thought, oh my God, she’s been through so much’, ‘Stressed because…I know the inevitable decline with dementia’ and ‘you suddenly realise you're in for the long haul, that your life probably isn’t going to be your own’.

Although a few carers struggled to accept the diagnosis, for most, this marked a point where life did change and as the bubble of normalisation burst, they worked out what happens next.

### Theme 4: What Happens Next?

This theme describes life after the diagnosis for the carer and PLWD. The first sub-theme – *‘The doors close’* represents the struggle carers faced to find help and support post-diagnosis. The second sub-theme – *‘Paying the price’* describes the impact of the efforts of carers to preserve the independence and autonomy of the PLWD. In the same way that the *‘Fear of talking about dementia’* meant, there were missed opportunities in the pre-diagnosis period, the third post-diagnosis sub-theme – *‘Still not talking’* describes how fear continued to prevent a conversation between the person and carer post-diagnosis.

### The Doors Close

In the weeks and months following the diagnosis many carers felt abandoned and alone. They described being given the diagnosis and then being left to work out what they needed to do by themselves, as the carer below describes,

“I assumed that there would be more help…I’ve had to carve, literally carve my way through finding out what’s out there…But there was a point right at the very beginning when there was nothing. I just couldn’t see my way forward. I didn’t know where we were going… we’d got the diagnosis but there wasn’t a clear route where, OK, now what do I do with this diagnosis?” (Carer 5)

In addition to the struggle of finding support, this carer questioned what to do with the actual label of the diagnosis. Whereas in earlier themes the diagnosis had the power to precipitate decline or change the person and carer, it appeared that post-diagnosis it did not have the power carers expected it would have to open doors and support them. Carers described ‘doors closing’ and compared the diagnosis to other medical diagnoses, with the expectation that the same level of help and support would be forthcoming. Others attributed the lack of interest to the incurable nature of dementia and the way society care for older people:

“I just thought, well this is a medical issue, what help can I get? And that was the wrong question. It was, what am I going to do? It took me quite a while to understand it’s all me, there isn’t anybody else out there… So you’re getting an awful lot of shut doors” (Carer 5)

“They [the doctor] lose interest, they’re not interested, there’s nothing they can do. One of the things that’s almost the worst thing…the doors close when you have dementia.” (Carer 8)

The perceived lack of available support was confirmed by the HCPs that carers were in contact with, ‘they referred me to…a dementia advisor but all he was really saying is, well, it’s going to get worse and worse and you’ll just have to live with it’, ‘I just said, so what’s going to happen now, and she [the doctor] basically said nothing. There wasn’t anything and she just said, well you can be referred to social services’.

This led carers to question the value of the diagnosis, ‘You can have a diagnosis but what does that mean you do as a carer’? and ‘We give people a diagnosis, so what?... My mother had a diagnosis, but it didn’t actually help’.

Carers described how they used the internet and books to find out more about dementia, what to expect and what support they were entitled to receive. A few carers wished they had known more about dementia to help them recognise what was happening, but most wished they had had more information about the progression of the disease and how to care for and communicate with the PLWD, so that they could have done more for the PLWD or put in place practical measures like power of attorney. Although some carers felt this information might have been helpful to know before their loved one developed dementia, as one carer explained, unless you know you are going to become a carer for a PLWD you may not see this information as relevant for you.

### Paying the Price

As in the time before diagnosis, carers’ ultimate concerns were still to protect the PLWD and preserve their independence for as long as possible, but there seemed to be a higher price to pay for this in the post-diagnostic period, both for the carer and the PLWD. Either due to the length of time carers managed before the diagnosis or the physical illness which had led to the admission, PLWD had significant needs when it came to discharge from hospital. Whereas previously carers were able to make decisions related to what they thought the PLWD would want, they now found themselves in a position where others had opinions about what they should do:

“the consultant said to me, if you take her home, it’s at your own risk…I think once you make that decision, that you’re going to go back home, that is when people are like, oh well, it’s up to you then.” (Carer 10)

“I had an enormous amount of pressure from the discharge nurse, no, no, please, put her into a care home, it’ll be much easier.” (Carer 12)

Carers thought that for HCPs, safety was prioritised over quality of life, but as the previous theme of ‘The doors close’ demonstrates, carers now felt less supported as they tried to preserve the person’s independence. Some carers went to great lengths to preserve the wishes of the person to be at home for as long as they could, sometimes in the face of strong opinions from family or friends that the person they cared for should move to a care home. But not all carers had the resources or were able to support the person at home as they would have wished, often resulting in feelings of guilt:

“it’s my fault, it’s because she’s been independent and we’ve kept her going and she’s been safe, she hasn’t used the system,…if she’d had a carer all this time and used the system and not been independent and proud and had people going to help her…it would have been in place for her…that’s very sad to me, that somebody who’s tried hard and been independent is now really paying a price for being that person.” (Carer 2)

The real conflict in the guilt this carer feels is evident. By resisting using ‘the system’ (social services) and providing care herself for the PLWD, she had been respecting their wishes. Now as the person needs to move to a care home, she reflects on whether the process would be less traumatic for the PLWD if she had already been ‘in the system’. But this would have meant not being ‘independent and proud’ and suggests that the two things – having help from social services and being independent and proud are not compatible.

There was also a price to pay in the relationship between the carer and the PLWD.

“I was very practical …Rather than the emotional side of it. But sometimes that’s the way you cope, isn’t it?” (Carer 10)

“And becoming a carer changes the dynamics, the power dynamics that, she didn’t like [that] but she was completely dependent.” (Carer 5)

Meeting the increasing needs of the PLWD could result in carers becoming task-orientated and at the same time facing resistance from the PLWD as they strived to retain their own independence. Although carers had been caring for the PLWD for a long time before the diagnosis, the diagnosis also formalised their role as ‘carer’.

### Still not Talking

In the same way that carers struggled to initiate conversations about their concerns in the pre-diagnosis period, very few spoke to the PLWD about their diagnosis afterwards.

Very few doctors disclosed the diagnosis to the PLWD. One carer described how the doctor, ‘…was trying to just speak in code so [PLWD] didn’t know’. This carer had to tell the PLWD the diagnosis themselves, taking an opportunity that presented itself when the PLWD showed them a magazine article about Alzheimer’s disease and expressed concern that they had many of the same symptoms.

But most carers did not discuss the diagnosis with the PLWD. This avoidance of the topic often resulted in carers being unsure as to whether the person knew the diagnosis, whether they wanted to know or had not understood the diagnosis. This again raised conflict in carers where on the one hand they hoped the PLWD did not know their diagnosis as it may invoke fear and on the other recognised how knowing might have helped the PLWD and might have relieved their fear, but as one carer said, ‘I didn’t know how to raise it’.

“I mean if you lay there and you think your mind’s going, you might really be frightened, whereas if someone says “oh mum this is old age dementia”, maybe you’d go “I knew it was something”… but you don’t get anybody talking to you about it” (Carer 3)

Despite recognising that knowing the diagnosis might help her mother and using this to reassure her mother in her imagined interaction, this carer had not spoken to her mother about it, possibly believing it was someone else’s responsibility to do this. A few carers worried that the person’s fear of the diagnosis might result in them harming themselves:

“she said, ‘well I’ll tell you what, that won’t be taking me. I won’t wait for that to get me’ she said. And I went, Mum, you could go another ten years before you get any worse...because I’m thinking, don’t top yourself… And so when she said it, that’s the thought I got…don’t do anything stupid, Mum.” (Carer 11)

“We never mention the word dementia and I don’t want anyone to mention [it]. We’ve watched programmes about Alzheimer’s and he says to me, ‘that must be so terrible, if anything like that ever happened to me, I’d do that’ he says (carer draws her finger across her throat). So Alzheimer’s for him is something that just happens to other people, fortunately he doesn’t have Alzheimer’s.” (Carer 8)

In addition to the PLWD’s fear, the carer’s own denial and fear is evident above as she does not want the word ‘dementia’ mentioned and makes comparisons to downplay the severity of the sub-type of dementia the PLWD has, compared to Alzheimer’s disease. It was apparent amongst all carers that by continuing not to talk about dementia, they were preserving some of the normalisation bubble that had offered protection in the pre-diagnosis period.

## Discussion

This study provides a unique insight into a group of carers of people who were diagnosed with dementia when they were not actively seeking help for a diagnosis. In common with carers in previous reviews of pathways to a dementia diagnosis, carers in this study moved through a cyclical process of noticing changes in the PLWD and seeking explanations for these. However, in these reviews carers and PLWD reach a point where increasing changes cannot be normalised and the observations of others, support from within the informal network, a pivotal event or crisis all lead to a decision to seek help.^6,8^ Despite increasing cognitive and behavioural problems and an awareness that these might be due to dementia, carers in this study were not actively seeking help for a diagnosis. Some did consult family and friends but either found their normalisations affirmed or their concerns dismissed, a factor recognised to delay diagnosis.^8,12^ Others experienced pivotal events such as the PLWD getting lost or burning saucepans that were noted in other studies to precipitate help seeking,^6^ yet these were still not enough for them to seek help. Instead, fear and stigma related to dementia appeared to be a strong influence on carer’s desires to protect the PLWD and their way of life. These concepts of fear and stigma transcended the pre-diagnosis period, as carers in this study continued not talking about dementia after the diagnosis with their focus remaining on preserving the independence of the PLWD. Carers had negative images of dementia that included loss of self and institutionalisation leading carers to protect and preserve the independence of the PLWD and to keep them at home for as long as they could. The concepts of protecting and preserving as key roles for carers are not new. An early study of family carers found that protecting loved ones from threats to their self-image, by minimising their difficulties and maximising their sense of independence, was more important to carers than providing instrumental care and that care aimed at preserving a sense of self for older people living in a care home was an important part of the family carer role.^29,30^ Two types of relative – the protective relative and the decisive relative were identified in a recent study of family member’s experiences as relatives of a PLWD, with the carers in the present study aligning closely with the protective relative.^31^ The protective relative provided practical support to the PLWD whilst, often unconsciously, hiding and denying that changes were happening in order to preserve the identity of the PLWD, protect their relationship with the PLWD and their way of life. In contrast, the decisive relative acknowledges the changes and initiates decisions and actions that involve help seeking. In the absence of a decisive relative, carers in this study did not consider seeking help.

However, the protective and preservative roles of carers have both been considered potentially damaging if maintained for a long period.^32^ Protecting the PLWD from what is happening to them can be seen as paternalistic and preserving the past fails to acknowledge that changes and adjustments may be needed to maintain self-esteem in the future.^32^ In protecting and preserving the PLWD, carers in this study were reinforcing the denial and stigma felt by both the PLWD and the carer, particularly as carers avoided talking about dementia. Many carers based their decision not to discuss their concerns or the diagnosis on what they thought the PLWD would want or had previously expressed. But by not talking to the PLWD they were potentially removing what they had sought so hard to preserve, which was the PLWD’s autonomy. Fear that a discussion with the PLWD may confirm their own concerns and lead to help seeking and a life changing diagnosis may be one factor that prevented discussions taking place. Fear of denial by the PLWD that places the carer in a position of seeking help without their consent may be another. Carers in previous studies have expressed the conflict they face in seeking help on another’s behalf and the potential this has to damage their relationship with the PLWD.^6^ Although this was not explicit for carers in this study, with hindsight, some carers wished the diagnosis had come earlier and that HCPs had initiated this, taking responsibility for seeking help out of their hands. Such opportunities to discuss concerns and initiate an assessment were not just missed by carers. Whilst a small number of PLWD had memory assessments in the months or years before their diagnosis, most were in contact with physical health services that did not initiate the assessment process. There are well recognised barriers to dementia diagnosis in primary care including time and financial constraints, diagnostic uncertainty, concerns about disclosure of the diagnosis and a lack of support services for PLWD, carers and HCPs.^14^ But stigma and therapeutic nihilism on the part of HCPs are also recognised barriers.^14,33^ However, GPs also describe a nuanced, cumulative process in which a dementia diagnosis in primary care evolves, to take account of the individual circumstances of each PLWD and their families.^34^ As the perspectives of HCPs were not explored in this study, this explanation of events should not be ruled out, although this contribution to protecting and preserving may also add to the stigma surrounding a dementia diagnosis. If a timely diagnosis is one which happens when a PLWD and their family express concerns and a need for help, this begs the question of what happens if they feel unable to discuss their concerns. Without a prompt or enquiry from a HCP about possible concerns, the PLWD and their carer may remain unsupported indefinitely.

The difficulties around discussing dementia also extended to the diagnostic process in this study. Despite a national case finding scheme in acute hospitals at the time of diagnosis for most of the participants, the process of assessment and diagnosis varied considerably. An evaluation of the case finding scheme in one area of England found that patients and carers were often unaware that memory assessments had taken place,^35^ a finding supported in this study. However, other carers had to use the opportunity to push for assessment and diagnosis, reflecting the variability with which the scheme has been implemented,^36^ but again suggesting that open conversations about memory concerns and assessment were not taking place. This continued with diagnosis disclosure. The practice of disclosing the diagnosis over the phone to the carer was common, leaving the carer unaware as to whether the PLWD had been told. A majority of people would want to be told their diagnosis if they had dementia, a trend that has increased over time.^37^ Although in some studies, carers of PLWD were less in favour of disclosure to the PLWD, despite wishing to know themselves if they had the disease.^22^ A review of dementia disclosure practice revealed that only 34% of GPs and 48% of specialists always told the PLWD their diagnosis, with 89% of GPs and 96% of specialists always telling the PLWD’s family.^38^ Concerns around initiating assessment, diagnosis and disclosure included when the person and/or their family were in denial and when families expressed concern but the person themselves was not worried. The practitioner’s own confidence and beliefs about dementia, the health system they worked in, the awareness and circumstances of the PLWD and stigma also contributed to decisions about whether to disclose the diagnosis.^38^ It is recommended that disclosing a diagnosis of dementia should be a process rather than a single event, with the opportunity to discuss the possibility of a diagnosis before it is confirmed.^22^ Whether an acute hospital admission is the most appropriate setting for this requires further research; however, what is clear is that it does require more open and honest communication between HCPs, PLWD and their carers.

It has been suggested that to be willing to diagnose and disclose dementia, HCP’s need to ‘appreciate the value of the diagnosis’ for the PLWD and their family (p1615).^37^ However, very few carers in this study described considering seeking help for a diagnosis, suggesting this was not an option they saw as being valuable to them, with some carers questioning the value of diagnosis after they had received it. This was largely due to the lack of support they felt in the post-diagnosis period, a finding supported in previous studies.^7^ The benefits of an early diagnosis may be outweighed if the PLWD and their carers are given a diagnosis but left without support.^39^ The biomedical model of dementia is the one most often portrayed in the media, with a focus on the progressive nature of the disease and the need to find a cure.^40^ Alternative models with a focus on living well with dementia tend to reside largely within policy and research and less within popular media.^41^ Without positive images of how PLWD and their families are supported to live well post-diagnosis, carers may see little benefit in seeking a diagnosis, which in their eyes, results in a stigmatising label rather than support. Self-stigma and label avoidance have been associated with reduced help seeking for mental health concerns^42^ and for carers in this study, the label of dementia had various meanings. It had the power to precipitate decline and make real the difficulties PLWD were experiencing in the pre-diagnosis period. It had the power to change the PLWD and the carer at diagnosis, was something to be concealed or seen as necessary for hospital discharge to happen. Post-diagnosis the carer’s role was formalised and although some carers felt that knowing the diagnosis would not benefit the PLWD, they hoped that the label would open doors and lead to support for them as a carer. As close family members and carers are most often the people who initiate help seeking for a dementia diagnosis, to encourage help seeking, the benefits of a diagnosis both to the PLWD and themselves as carer need to be clear.

## Strengths and Limitations

This is the first study to consider the dementia diagnosis help seeking process from the perspective of carers who were not actively seeking help and allows an insight into factors influencing help seeking decisions in a group of people who are often difficult to identify and support. The role of stigma in preventing discussion about dementia suggests this may be an important factor preventing help seeking, that extends to HCPs as well as carers and PLWD.

A limitation of this study is the absence of the voice of the people living with dementia. The original study aim was to explore the perspectives of people diagnosed during or following a hospital admission in addition to their carers, but we were unable to recruit any PLWD to the study. It is a criticism of dementia literature, and that focused on stigma and dementia, that the carer voice dominates these narratives and as such may serve to reinforce the stigma experienced by people living with dementia.^43^ But by understanding the experiences of these carers, and particularly their difficulties in talking about dementia, future research and development should aim to support carers to talk about their concerns and in turn encourage conversations and the voice of the PLWD to be heard.

The role of HCPs in initiating discussions about dementia concerns was an important factor in the findings of the study, although this was only explored from the perspective of carers. It is possible that HCPs did express concerns to the PLWD or their carer that were not subsequently followed up. Further research should aim to understand the experience of HCPs in discussing concerns about dementia with people with symptoms and their families.

The small sample size and restricted geographical area in which recruitment took place, is a limitation of the study. This limits the generalisability of the findings. However, carers from across London, in areas with differing diagnostic practices took part. This suggests that the difficulties they had talking about dementia and challenges post-diagnosis were not isolated to carers in a small area and further research is needed to determine whether carers in other areas of the UK have similar experiences. Stigma has been recognised as a barrier to participation in dementia research and may have contributed to the difficulty in recruiting carers for this study, particularly in the days or weeks following diagnosis.^44^ Adjusting to a diagnosis, in addition to managing the mental and physical frailties of a PLWD during an acute hospital admission are recognised challenges for recruiting carers to research studies.^45,46^ Indeed one carer from the hospital who initially expressed an interest in participating later declined participation due to the difficulties they were having arranging discharge for the PLWD. It is also possible that the carers recruited from JDR may differ from other dementia carers. For some of these carers, the time between the diagnosis of the person they cared for and their interview was a number of years. This could potentially result in some recall bias when discussing their experiences. However, these were significant events in their life, that they were likely to remember and the experiences of the carers of people diagnosed 9 years prior to interview did not differ greatly from those diagnosed weeks prior to interview, suggesting that recall bias did not significantly impact the findings of the study. The small number of carers from JDR eligible to participate may also reflect stigma related to dementia and its ongoing impact on the ability of carers to talk about dementia.

## Conclusion

With policy imperatives and interventions to increase timely diagnosis of dementia, particularly in the UK, understanding the experiences of those who do not seek help is increasingly important.

Increased public awareness of dementia meant that most carers considered dementia the possible cause for the difficulties the PLWD was having. But increased awareness may lead to increased fear.^47^ Stigma related to images of dementia as a disease that takes away independence and identity prevented discussion about dementia between carers and the PLWD in this study. This was exacerbated by a lack of open discussion about memory concerns between HCPs and carers as they came into contact with health services.

Carers chose to create a bubble of normalisation around the PLWD to protect and preserve their independence rather than discuss their concerns and seek help. The lack of support post-diagnosis serves to further reinforce negative images that ‘nothing can be done’ for a PLWD. If we are to encourage people to seek help for a dementia diagnosis then positive images of people living well with dementia and being supported to maintain their independence and autonomy need to be the mainstream, rather than the catastrophic views often portrayed in the media.^48^ Although, service provision would need to match expectations for the benefit of diagnosis to be valued. Future interventions targeted at supporting family members to discuss concerns about dementia with their loved ones may result in a diagnostic process that is governed by the person and their carer and results in a diagnosis that comes at the right time for them. Health care professionals also need the confidence to talk openly about dementia with patients and their families to encourage those who have concerns to share these and seek help earlier.

The COVID-19 pandemic presented considerable challenges in the move to remote provision of memory assessment services, delivery of dementia diagnoses and in offering post-diagnosis support.^49^ Whilst accessing these services remotely will have suited some people it may also have further deterred some carers from talking about concerns with the person they care for and initiating an appointment for assessment. Limited in person contact with primary HCPs may also reduce cognitive problems being identified and discussed. A challenge for the immediate future will be to ensure that people do feel able to raise concerns about their memory with their GP and access a memory assessment if needed. But an increasingly proactive approach to identifying people with possible dementia may be needed in the longer term to ensure that people are supported to access a diagnosis and post-diagnosis support when the time is right for them.^50^

## Supplemental Material

sj-pdf-1-jgp-10.1177_08919887211060018 – Supplemental Material for The Bubble of Normalisation: A Qualitative Study of Carers of People With Dementia Who Do Not Seek Help for a Diagnosis Click here for additional data file. Supplemental Material, sj-pdf-1-jgp-10.1177_08919887211060018 for The Bubble of Normalisation: A Qualitative Study of Carers of People With Dementia Who Do Not Seek Help for a Diagnosis by Michelle Parker, Sally Barlow, Juanita Hoe and Leanne M. Aitken in Journal of Geriatric Psychiatry and Neurology
