# Supplementary material for: The Bubble of Normalisation: A Qualitative Study of Carers of People With Dementia Who Do Not Seek Help for a Diagnosis
Source: J Geriatr Psychiatry Neurol. 2021 Dec 24;35(5):717–32. doi: 10.1177/08919887211060018 (PMC9386763; doi:10.1177/08919887211060018)
Supplement: sj-pdf-1-jgp-10.1177_08919887211060018 – Supplemental Material for The Bubble of Normalisation: A Qualitative Study of Carers of People With Dementia Who Do Not Seek Help for a Diagnosis [file sj-pdf-1-jgp-10.1177_08919887211060018.pdf]

Theme	Sub-theme	Illustrative Quotes
Theme 1 Normalisation as a bubble	Explaining to normalise	***Making comparisons to existing knowledge***<br>“sometimes I thought it was like an act…to get our attention…seemed to be almost like she knew what she was doing…. so I’m thinking she’s not got dementia she’s quite shrewd…” (Carer 3)<br>“I was worried…because I’d seen people with dementia, that we were going to have a real problem down the line…she never ever lost her temper, which you sometimes hear about…she never became aggressive, never.” (Carer 1)<br>“But he didn’t have that before, so it’s quite sudden…it wasn’t sort of a gradual thing, which is what I’ve always thought, and from other people that I know” (Carer 4)<br>**Advice from family and friends**<br>“so I spoke to friends and people but nobody I know has…experienced a relative with dementia. So it was very like, oh it’s old age, this is what happens to people, and, you’ve got to expect it, she’s getting on a bit.” (Carer 10)<br>“I said to my brothers and sisters don’t laugh because I think this is a condition that is not really funny…I just felt they weren’t in the real world, they couldn’t see what was happening in front of them.” (Carer 1)<br>“I did mention it to other people in the family like my sons, my nieces, nephews, to watch out for her…Some of them turned around and went, well… she is 90. I just think they think everyone of a certain age will forget things and so that’s all it is, you know?” (Carer 11)<br>“My mother and I had discussed it…my mother was quite astute and, yeah, so she knew” (Carer 7)<br>**Negative views of dementia**<br>“…from my point of view, I was in denial…no illness is great, but dementia just robs you of so much” (Carer 10)<br>“I’ve seen people with dementia and it’s sad…because those that I had seen had no one to care for them. I’d

		seen people with dementia put in a home, negative things" (Carer 1)<br>"I wouldn't like to be, if I get dementia in the future people just saying, oh yes she's got dementia. It's quite a label isn't it?" (Carer 5)<br>"Well, it's degenerative, isn't it? And you don't know where it's going to go. You don't know when the person will recognise you." (Carer 7)<br>"a diagnosis of Alzheimer's or dementia is such a devastating thing" (Carer 8)<br>"people are frightened by dementia… they're frightened cos it could happen to any of us and I think it's quite shocking to see… old age isn't pleasant for anyone is it… as you are older that you know it could happen to you and you will lose who you are as a person… its an awful thing…it makes you very vulnerable" (Carer 2)
Theme 1 Normalisation as a bubble	To protect and preserve	**Balancing independence with increasing needs**<br>"They showed me around [day centre] but she wouldn't have fitted in there at all … she wasn't anywhere near this stage, she was a lot better, independent, walking and I could see the expression on her face…it was a bit going to be like a mother or a parent taking their child for the first time to school and then leaving them there and…I assured her…that's not the place for you" (Carer 1)<br>"she's just always been a very independent woman that has gone everywhere, done everything… I just thought maybe she should be getting some help, but she's so independent." (Carer 11)<br>"She was quite an independent lady…and always had looked after herself…she was quite proud as a person and a strong personality, you know she'd say Oh I'm fine, I like where I live and I don't need any help and… you do my jobs… I tried to put it to her someone would come in and it would be nice, you know, to help you just get dressed and everything, it used to just upset her more than anything so I didn't really push her" (Carer 2)<br>"my mum was a very independent kind of lady, she could, she would only ask you to do something if she really,

		really couldn't do it" (Carer 6)<br>"both my parents… wouldn't want to burden me or my younger sister or trouble us or worry us untowardly or anything like that… I think a bit of embarrassment… again that's personal pride type thing, I suppose" (Carer 9)<br>"the default with the family is to protect someone. So nobody wants to know, nobody necessarily needs to know except of course if there's an issue with driving… you know in a way it's all about people's rights and you know we don't want to take too many rights away from someone who has dementia."…"But I think partly this is to protect me, partly was to protect him, partly it was to protect our way of life." (Carer 8).<br>"a move was on the agenda. And she put off, put off, put off… and was fighting us until, I think even she realised. But the move itself…precipitated a dip in her dementia and one which I don't think my brother and I have ever recovered from, in terms of guilt" (Carer 5)
Theme 2 Missed Opportunities	Fear of talking about dementia	**Carer concerns dismissed**<br>"she just [said], oh no, I'm all right, it's just old age. She wasn't worried about it." (Carer 10)<br>"I did speak to her…I'd say no I'm worried you might need some help and everything, but she's always been independent so it's not something she wanted." (Carer 2)<br>"just said, maybe he should have some kind of testing or go to the doctor about it and he wouldn't" (Carer 7)<br><br>**PLWD concerns dismissed**<br>"he used to say, when he was getting, becoming a bit forgetful, but no more than anybody else really, oh I must have dementia, I must. I said dad, you don't have dementia, and then he went to the doctor's once and said oh, I keep forgetting things, I must have dementia." (Carer 4)<br>"I remember him once saying to me, something terrible has gone wrong with my brain…. But however much it

		might have gone on inside him, he didn't really manifest it like that and I tried to be, not to engage with it at all" (Carer 8)<br><br>**PLWD concerns not shared**<br>"he used to keep a diary sometimes of events…the way he felt about things…and he said 'something's happened that's changed everything, I'll keep you up to date' in other words talking to the computer, I'll update it and there's no other entry." (Carer 8)<br>"he was concerned about his health and keeping a log but wasn't necessarily sharing it with people" (Carer 9)
Theme 2 Missed Opportunities	Contact with Health Professionals	**GP awareness**<br>"because she was on warfarin, she was going to see a doctor every month. And they had not said anything about her mental condition. The only time I was allowed to take her to the GP was when she couldn't walk…I did that for a couple of visits, but nobody suggested anything" (Carer 10)<br>"I accompanied her, and the doctor refused to give her more. She was on low dose diazepam…A, because she'd been falling, B, because it was diazepam, C, because the doctor I think realised this wasn't anxiety, this was dementia." (Carer 5)<br><br>**Memory assessments**<br>"the doctor referred us to the Memory Clinic…and we went for a couple of appointments…and he wasn't [diagnosed with dementia]. So… I don't know, I just think he was looking for a bit of sympathy" (Carer 4)<br>"a nurse came round, and gave her a dementia test… which she didn't pass, but only because she didn't understand… because she was speaking English… she said it's not dementia" (Carer 1)<br>"we went a couple of times to the memory clinic…They just talked to her and did a few tests…but nobody really

		said she had dementia." (Carer 10)<br>"usually there was ten questions…I was wondering whether she didn't know or sometimes she was a bit stubborn, she didn't want to speak…I thought, I'm sure even if they asked me sometimes I will probably not remember" (Carer 6)<br><br>**Fragmented system**<br>"I taped her in her chair, how bad she was, so when the social worker came I said look I've got to show you this because… nobody's believing how bad mum is…[Interviewer: Did the social worker suggest her being referred to a memory clinic or seeing the doctor about her behaviour?] No she used to sort of come round to make sure she was managing at home" (Carer 3)
Theme 3 Diagnosis - Bursting the Bubble		**Carers initiating assessment**<br>"a lovely consultant there…she did a brief capacity test on her because I mentioned it to her that I was concerned. And she got things wrong, so she said, oh, this is something we'll have to follow up while you're in here. But nothing happened. She was in for three days, and I was really hoping something would get the ball rolling then but no it didn't" (Carer 11)<br>"I kept saying that I'm really worried about her…then I saw the doctor as much as I could and then I think they said to me that there'd be someone [to assess PLWD]" (Carer 3)<br><br>**Reaction to diagnosis**<br>"I felt really…that they just needed to diagnose and call it something, that's how we felt…they needed to have the diagnosis to say to a care home or a nursing home look, this man's got whatever, and that's because they didn't really have one. So I almost felt it was, well we don't really know, so we're going to call it this, that's how

		we felt” (Carer 4)<br>“So when he said quite categorically that she has Alzheimer’s I was slightly shocked, defensive. But that labelling I think gets in the way also…that even if you know that this is the right diagnosis you're going to keep that to yourself in certain contexts.” (Carer 5)<br><br>**Timing of diagnosis**<br>“I don’t think mum would have taken it earlier, if I’m honest” (Carer 11)<br>“I don’t think it was right for either of us. I think it was extremely abrupt and I was in a real state. And so was she. And I think, I actually think that it wasn’t properly considered,” (Carer 12)<br>“Oh overdue I’d say, yeah, I needed that [diagnosis] well before it [hospital admission], we needed to know what was the matter with her” (Carer 3)<br>“I think if we’d had a diagnosis, say, a year earlier, it would have been better for us. It probably wouldn’t have made any difference to him” (Carer 7)<br>“I’d have preferred it earlier. Earlier with either him making me aware of it to get him looked at earlier and then I think, yeah by the time then we suspected… So, yes, I do wish it was done earlier, because I could have taken more preventative measures at home” (Carer 9)
**Theme 4 What happens next?**	**The doors close**	“At least when someone gets a cancer diagnosis there’s some understanding of what the disease is going to do… and it’s just as devastating, and it’s not treated like it’s an illness either…if you have any other illness, then your care is paid for. I mean I think it’s treated like mental health. And that’s not treated very well.” (Carer 7)<br>“it’s on par with being diagnosed with cancer but of course in some cases its infinitely worse” (Carer 8)<br>“it was in the paper today how bad we are for looking after our elderly… just can’t as a society…I think we’re a bit lacking in that department for looking after them” (Carer 3)

		"even with a diagnosis it doesn't necessarily help the person who's diagnosed if they don't want to know. Even if you're a carer knowing isn't, … it didn't help me with knowing what I should do. You can have a diagnosis but what does that mean you do as a carer?" (Carer 5)<br>"So, we give people a diagnosis, so what? And then we say, and you're going to get worse and worse and you'll die and there's nothing we can do about it, except stick you in a home and wait for you to die." (Carer 12)<br><br>"There wasn't anything helpful and it wasn't until I'd got over the anger and shock and I'd started Googling, that I could find things that might help." (Carer 12)<br>"I do think once we'd had the diagnosis, it would have been really nice if someone had come and had a chat with [PLWD] and me and talked about exactly how [he] was and how best for us to talk to him. It's lucky that I came across this book that I found helpful, but I think if I hadn't, that would have been not so good." (Carer 7)<br>"I think it would have been helpful for me as a carer to be educated about dementia" (Carer 10)<br>"saying how would that help somebody, is assuming that they have a very clear idea that they're going to be the carer. And it wasn't clear…if somebody had said, look right from now on you're going to be the carer, she's not going to get better, you need to know about this, that would have helped me. But at the time I didn't know I was going to be a carer." (Carer 5)
Theme 4 What happens next?	Paying the price	"It used to upset me a lot when friends, relatives used to say, oh, you're not going to cope, you're doing yourself harm, why don't you take her into a care home, and as soon as they used to mention that it used to really upset me and I'm glad I didn't listen to them anyway." (Carer 6)<br><br>"a lot of people say to me "Oh you should think about a residential.." I think what they were doing they were

		projecting their problems and so because they couldn't deal with it and they were saying you can't do this, you've got to think of her and don't be a martyr, you're doing this" But I think…you see not many people can cope really and it was tough but I coped and I'm glad I've done it now, we're coming to the very end of it all I think" (Carer 1)<br><br>It's a really hard thing to live with. So I think I've got a lot of guilt from that point of view, that she's ended up where she is, and it's not where she wants to be" (Carer 10)<br><br>"she wasn't happy a lot of the time and I wanted her to be. And I think I could have made her happier for longer. I didn't give her enough attention and I was just so exhausted all the time. And I'm really glad I did what I did but I wish I'd done more." (Carer 12)
Theme 4 What happens next?	Still not talking	"I don't know if he would understand, and if he did, I don't, I don't know, to be honest, about that" (Carer 4)<br><br>"I hoped she didn't hear it. She was going deaf… I think in a way it might have been a relief because she would then have known what was making her fuzzy. I think she might, if I had said, we're going to fight this one, she might have done it" (Carer 12)<br><br>"She didn't really understand it to be honest. They said it to her, but I don't really think, because even today, she doesn't know she's got dementia… But I don't think it would have helped my mum, to know really what she was suffering from…I think it would have frightened her." (Carer 10)<br><br>"My mother has been in denial about dementia. She got a diagnosis but literally it was like she didn't hear it. She

		didn't want to hear it, won't hear it" (Carer 5)<br><br>"I think I've taken another little bit of her confidence away by telling her if I'm honest, because since we had that conversation, she has got worse… which I'm upset about because I don't want to do that." (Carer 11)
